# Oral Health Behaviours, Knowledge, and Literacy of Expectant Mothers: A Cross-Sectional Study among Maternity Ward Patients

**DOI:** 10.3390/ijerph191811762

**Published:** 2022-09-18

**Authors:** Ewelina Chawłowska, Monika Karasiewicz, Agnieszka Lipiak, Rafał Staszewski, Mateusz Cofta, Maria Biskupska, Bogusz Giernaś, Agnieszka Zawiejska

**Affiliations:** 1 Department of Preventive Medicine, Poznan University of Medical Sciences, 60-781 Poznan, Poland; 2 Department of Hypertension, Angiology and Internal Medicine, Poznan University of Medical Sciences, 61-848 Poznan, Poland; 3 Department of Medical Simulation, Chair of Medical Education, Poznan University of Medical Sciences, 60-806 Poznan, Poland

**Keywords:** health behaviours, oral health literacy, oral health knowledge, health promotion, pregnant women

## Abstract

Maternal knowledge, literacy, and behaviours in the area of oral health may greatly influence the risk of caries and the oral health status of children from the youngest age. Thus, the aim of the study was to investigate paediatric oral health knowledge and literacy as well as maternal oral health behaviours and outcomes among expectant mothers. A cross-sectional study was undertaken among 400 pregnant inpatients aged 17–48 years (mean age 29.5 ± 5.3 years) in 31 public maternity wards in the Wielkopolska region, Poland. An anonymous, self-designed questionnaire was prepared on the basis of current oral health recommendations. Pregnancy complications were reported by 195 (48.8%), and permanent tooth extractions by 158 (39.5%) women. Knowledge and literacy scores were associated with, among other things, maternal education, selected oral hygiene practices, and reported extractions of permanent teeth. Although participants had some correct information regarding oral health, they had insufficient awareness of caries as an infectious disease and of the appropriate timing for the child’s first dental visit. Their self-assessment of oral health status and belief that they were under dental care tended to be overly optimistic, given their self-reported outcomes. These aspects should be considered in future health education efforts among expectant women.

## 1. Introduction

Due to an interplay between shared genetic and environmental factors, oral health (OH) status seems to run in families [1,2,3,4]. Children’s oral health outcomes were found to be associated, among other things, with their caregivers’ oral health literacy, knowledge, and behaviours [3,5,6,7,8].

There have been a number of attempts to conceptualise [9,10,11,12] and examine [13,14,15,16] interactions between general health outcomes, literacy, knowledge, and behaviours. The notion that has recently gained prominence in relevant research is health literacy (HL), i.e., “the degree to which individuals have the capacity to obtain, process, and understand basic health information and services needed to make appropriate health decisions” [17]. Moreover, due to the high prevalence of dental caries worldwide [18], there is increased interest in the relationships between oral health outcomes, literacy, knowledge, and behaviours [19,20,21,22,23]. It has been suggested that oral health knowledge and literacy (OHKL) strongly influence oral health status [24], particularly in paediatric populations [8,25], which is why these aspects need to be examined before any public health interventions are considered.

Poland was reported to have had the highest early childhood caries incidence rate for 2019 in the European Union [26]. To address this alarming issue, it is crucial to reach out to and counsel mothers—facilitators of children’s OH—as early as possible. Educational interventions directed at children’s OH are even recommended during pregnancy [27,28,29,30,31,32,33]. The interventions should be preceded by exploring expectant mothers’ knowledge; however, studies that investigate OH knowledge among pregnant women in Poland are scarce [34,35,36,37,38,39]. The study presented here is an attempt at addressing this scarcity in a novel way. Namely, it investigates pregnant women’s knowledge with reference to three modifiable factors of caries aetiology, which are the most important from the point of view of early childhood caries prevention. In addition, it presents a preliminary analysis of women’s paediatric oral health literacy. While health literacy itself had been studied in Polish populations [40,41,42], the concept of oral health literacy among pregnant women has not been explored in Poland. Finally, this study investigates interactions between oral health behaviours, knowledge, and literacy of Polish expectant mothers.

## 2. Materials and Methods

### 2.1. Participants

A cross-sectional survey-based study was carried out among expectant women of the Wielkopolska region of Poland. The survey took two weeks and was conducted by qualified interviewers in maternity wards of 31 public hospitals by means of paper-and-pencil personal interviewing. The interviewers had been pre-instructed by the team of questionnaire designers on how to provide full anonymity and comfort during the interviews. All participants were informed that participation in the study was fully voluntary and anonymous and that the data collected would be used for scientific purposes only. The respondents had an opportunity to ask questions during the data collection process or cancel their participation and withdraw from the study at any point before the questionnaires were collected. Informed consent was obtained from study participants. The Bioethics Committee of the Poznan University of Medical Sciences confirmed that the study did not constitute a scientific experiment and, as such, did not need ethical approval in accordance with Polish law and good clinical practice guidelines (decision no. KB-427/22).

Given the 5-year annual average of 38,162 live births in Wielkopolska [43], the mean number of births per a 2-week period was 1468 (38,162 births a year/52 weeks). Thus, we determined the minimum sample size to be 304 at a confidence level of 95%.

### 2.2. Measures

A questionnaire was prepared in the Laboratory of International Health at the Department of Preventive Medicine of the Poznan University of Medical Sciences. Although the questionnaire was self-developed, its questions were prepared on the basis of a literature review of recommendations regarding oral health practices for paediatric and adult populations [27,44,45,46,47]. The instrument was consulted for face and content validity with five expert judges in the fields of oral health, public health, and obstetrics, each with at least ten years of professional experience. The resulting tool aimed to gather respondents’ demographics (age, residence, education, number, and age of children), pregnancy data (presence of pregnancy complications), and details of the use of dental care services (regularity, economic aspects). It also contained a section devoted to respondents’ knowledge, literacy, behaviours, and outcomes. More specifically, this section consisted of the following areas:

(1) Paediatric oral health knowledge and literacy area comprising questions and statements related to preventing dental caries in children. This area consisted of:
  - The oral health knowledge scale (OHK16), consisting of sixteen true/false questions in three sections, related to three modifiable factors of caries aetiology [41]: (a) the bacteria section, concerning the infectious character of dental caries; (b) the substrate section, concerning a cariostatic diet; and (c) the time section, involving proper oral hygiene to shorten the exposure to bacterial products.
  - Oral health literacy questions (OHL2) comprising one open-ended question asking when the child’s first dental check-up should be sought, and one multiple-choice question asking where the woman obtained information about the proper oral hygiene of her child.
(2) The maternal oral health behaviours area consisted of single and multiple-choice questions.
(3) The maternal oral health outcomes area contained three questions (to check if the reported behaviours translated into perceived oral health outcomes).

### 2.3. Data Analysis

Statistical analyses of the data were performed with the Polish version of STATISTICA 13 (TIBCO, Palo Alto, CA, USA), R version 4.2.1, and R studio version 2022.2.3.492 (RStudio, PBC, Boston, MA, USA) [48]. The descriptive analysis was carried out and a number of statistical tests were performed to examine the relationships between variables. Depending on the kind of data collected, the following tests were used: chi-squared test, Mann–Whitney U test, ANOVA Kruskal–Wallis test, and Spearman’s rank correlation coefficient.

To investigate the relationships between the outcomes and explanatory variables in the multivariate analysis, a stepwise logistic regression model was fitted for each of three OH outcomes. The explanatory variables introduced to the models included maternal demographics, OH behaviours, OH literacy, and OH knowledge scores expressed as percentages of correct answers for the total OHK16, and separately for the bacteria, time, and substrate sections of the scale.

In all of the analyses carried out, *p* values < 0.05 were considered statistically significant.

## 3. Results

The study group consisted of four hundred pregnant inpatients aged 17–48 years (mean age 29.5 years, ±5.3). Most of the respondents either had higher (41.5%) or secondary education (40.3%). Nearly half (47.5%) lived in the country. A vast majority self-assessed their economic status as good (95.3%). A third of the participants were in their first pregnancies. Pregnancy complications were reported by 48.8% of the women. Approximately half (50.6%) reported being in the ninth month of pregnancy, which might mean that at least some of them were hospitalised owing to imminent delivery. The characteristics of the study group can be found in Table 1.

### 3.1. Paediatric Oral Health Knowledge and Literacy

**Figure 1:**
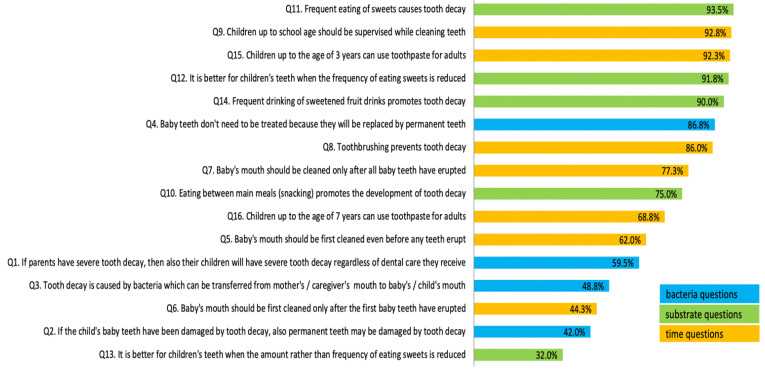
Percentages of correct answers to paediatric oral health knowledge questions (OHK16).

Paediatric oral health knowledge (OHK) in the study group was assessed using the self-developed OHK16 scale, comprising sixteen questions across three sections: (a) a bacteria section with four questions, (b) a substrate section with five questions, and (c) a time section with seven questions. The total OHK16 score was, on average, 11.4 points (±2.6), denoting mean correctness of 71.4%. The group’s correctness scores in sections referring to aetiology factors were as follows: bacteria 59.2% (±26.5%), substrate 76.4% (±20.2%), and time 74.7% (±20.6%; see Figure 1).

The lowest scores were observed in the bacteria section. Only 42% of women agreed with the statement “If the child’s baby teeth have been damaged by tooth decay, also permanent teeth may be damaged by tooth decay”. The statement “Tooth decay is caused by bacteria which can be transferred from mother’s/caregiver’s mouth to baby’s/child’s mouth” was identified as true by 48.8%. While the bacteria-related statements turned out to be the most difficult for expectant mothers to answer correctly, it should be noted that they were also the longest in terms of their word counts in Polish.

This area also included two oral health literacy questions (OHL2). The first one asked when the child should undergo the first dental check-up. This question was answered correctly (i.e., up to the age of twelve months) by 19.5% (*n* = 78) of our respondents. The most common responses were “at two years of age” (*n* = 148; 37%), “at three years of age” (*n* = 94; 23.5%), and “at four years of age” (*n* = 36; 9%). The ages of five and six years were each indicated by 4.75% (*n* = 19), and seven years was indicated by 1.5% (*n* = 6).

The second literacy question referred to the woman seeking information about the child’s OH and hygiene. Notably, only 53.7% (*n* = 215) of respondents reported that during or shortly before becoming pregnant they obtained any paediatric OH information from sources, such as the internet (*n* = 87; 21.8%), a dentist (*n* = 84; 21%), paediatric magazines (*n* = 60; 15%), an obstetrician–gynaecologist (*n* = 46; 11.5%), family members or friends (*n* = 38; 9.5%), a community midwife (*n* = 31; 7.8%), or antenatal classes (*n* = 20; 5%).

### 3.2. Maternal Oral Health Behaviours and Outcomes

Next, the survey investigated the women’s reported OH behaviours, OH self-assessment, and reported outcomes (Table 2). Relations between these aspects were also analysed. Regarding maternal behaviours, most women stated that they brushed their teeth regularly (81.1%) with fluoride toothpaste (96%). Interestingly, while 90.3% of respondents said they were under dental care, only about half (54.4%) of all respondents had been to a dental visit within the previous six months (see Table 2). On the other hand, nearly all (98.17%) of the women who reported visiting a dentist within the last half-year expressed a belief that they were under dental care, while only 80.77% of the remaining women expressed such a belief (*p* =0.00001).

About a third of respondents (30.2%; *n* = 121) stated that their economic status had been a barrier to using dental care services at least once. The responses to this question were associated with permanent tooth extractions in the past (*p* = 0.00001) and with self-assessed OH (*p* = 0.00001). The women who reported such a barrier also more often mentioned toothache as a reason for their latest dental appointment (*p* = 0.04769). However, financial barriers were not reported more often by women who had last visited a dentist over six months before when compared to those who had seen a dentist more recently (*p* = 0.12899).

The majority of women rated their teeth as healthy or rather healthy, and the self-assessment of OH was associated with self-reported economic status (*p* = 0.00475). About one in five women assessed their OH status as unhealthy/rather unhealthy/unknown, even though 39.5% of respondents had experienced an extraction due to caries or periodontal disease, and 32.5% mentioned pain as a reason for their last dental visit. Still, it could be said that the self-assessed OH was reflected in self-reported OH outcomes: the group reporting healthier teeth included more women without extractions (65.33% vs. 40.26%; *p* = 0.00005) and more often mentioned prevention as a reason for the last dental visit (65.63% vs. 37.66%; *p* = 0.00001) than the group with poorer self-reported OH.

As for reported outcomes, the women who had undergone extractions were also more likely to give pain as a reason for dental care use than the women without extractions (49.37% vs. 33.47%; *p* = 0.00149). However, the two groups did not differ with respect to the dates of their last dental visits (*p* = 0.31519) or the belief that they were under dental care (*p* = 0.37092).

Interestingly, only one association was found between the reported outcomes and behaviours, namely between being under dental care and pain as a reason for the last dental visit (*p* = 0.02523). None of the other OH behaviours differentiated the outcomes.

### 3.3. Paediatric Oral Health Knowledge and Literacy vs. Demographics, Behaviours, and Outcomes

Another investigated aspect involved the associations between respondents’ paediatric OH knowledge and literacy on the one hand, and demographic data, reported OH behaviours, and reported OH outcomes, on the other hand (Table 3). It was found that age, education, and the number of children differentiated the total knowledge scores in OHK16. The total OHK16 scores were also linked with certain behaviours (regular toothbrushing, fluoride toothpaste use) and outcomes (self-assessed OH, lack of extractions, visiting a dentist for a reason other than pain).

When it comes to the literacy questions (OHL2), the awareness of the timing of the child’s first dental check-up was associated with three variables: the number of children in the family, having a preschool child, and past extractions. The responses to the second question—obtaining information on the child’s OH during or shortly before pregnancy—were associated with having a preschool child and brushing teeth regularly.

### 3.4. Multivariate Analysis of the Associations between Self-Reported Outcomes and Participants’ Characteristics

Multivariate analysis determined that participants indicating economic status as a barrier to dental care or not being under dental care were less likely to self-assess their oral health status as good (Table 4).

Next, the analysis identified three participants’ characteristics associated with the elevated risk of permanent teeth extractions: economic status as a barrier to dental care, lower levels of education, and maternal age. On the contrary, women living in small towns were less likely to report this outcome compared to those living in big cities (Table 4).

Finally, the analysis showed that participants with lower levels of education were more likely to report pain as a reason for the last dental appointment. In addition, being under dental care was related to an approximate 60% reduction in this risk. Moreover, participants with higher scores in the bacteria section of the OHK16 were slightly less likely to report this outcome (Table 4).

## 4. Discussion

### 4.1. Paediatric Oral Health Knowledge and Literacy

The results of this study suggest that the paediatric OH knowledge and literacy of expectant mothers may be insufficient for making appropriate well-informed choices, which may later translate to neglect of OH and a higher risk of poorer maternal and paediatric outcomes. According to most Polish studies in similar populations, OH knowledge and skills are limited, inadequate, or none [34,35,37,39]. In contrast, one recent study with 106 respondents (reached through online groups of pregnant women) had areas where the results were more optimistic. For example, 82.1% of its respondents knew that a baby’s mouth should be cleaned even before the first teeth appeared (vs. 62.0% in our group), and 80.2% knew that frequency rather than quantity of sweets consumption increases caries risk (vs. 32.0% in our group) [38]. Although the studies are difficult to compare due to methodological differences, they share a conclusion that OHK among expectant mothers in Poland requires more effective educational interventions. However, it should be stressed that the educational needs in these populations have not been analysed in terms of broader knowledge areas, such as bacteria, time, and substrate sections used in this study. Such an approach could help to determine which OHK area is the most neglected and, thus, lead to more effective counselling. Similarly, the concept of OHL among expectant mothers has not been investigated in Poland despite the growing recognition of health literacy as an important factor associated with health behaviours and outcomes. In addition, the questions of the survey tool were based on international recommendations on caries prevention. That is why it could be treated as the first step towards constructing an instrument for international comparisons, for example, between countries with difficulties in accessing OH education, particularly those with similar caries prevalence (Romania, Hungary, Bulgaria, Latvia, Croatia) [49,50].

In the present study, the average score of correct answers on the OHK16 scale was 71.4%. The incorrect responses in the bacteria section influenced the total score the most. During the data analysis, respondents’ scores were higher in the sections containing shorter statements. The bacteria section had, on average, 14.5 words per statement, the substrate section had 9.2 words per statement, and the time section had 10 words per statement (with section scores of 59.2%, 76.4%, and 74.7%, respectively). This may indicate that the differences between section scores were connected with health literacy, in particular with one of its aspects referred to as functional literacy [51], which is defined as basic reading and writing skills enabling an individual to function effectively in health-related contexts. The relation between OH knowledge and literacy requires further in-depth research. On the other hand, the low scores in the bacteria section may suggest that expectant mothers had insufficient awareness of the infectious character of tooth decay, especially of themselves as a possible source of infection (Q.3 = 48.8% of correct answers) and of the relation between caries in primary and permanent dentition (Q.2 = 42% of correct answers). At the same time, 86.8% of respondents knew that decay in primary dentition should be treated (Q.4). To compare, 71% of the surveyed Croatian [52] and 90% of Portuguese [53] expectant mothers believed that primary teeth should be treated. Notably, in the latter study, only 10% of respondents knew that caries had infectious nature. Such results, as well as our findings, may suggest that women recognise the importance of treating deciduous teeth for reasons other than preventing the transmission of cariogenic bacteria to secondary teeth. In addition, research shows that women may not fully understand the mechanism of transmitting caries between family members. For instance, only 36% of pregnant patients surveyed in the USA knew that a mother could infect her child [54]. This may indicate that educational interventions stressing the aetiology of caries could lead to a better understanding of the health problem and possibly motivate mothers to prevent infections [54] by eliminating such practices as cleaning a dummy with the mother’s mouth or sharing feeding utensils.

It could be expected that patients lacking knowledge of the role of bacteria in dental caries may also be unaware of the importance of regular oral hygiene practices [54]. In the presented study, the average percentage of correct answers in the relevant (i.e., time) section of OHK16 was 74.7% (±20.6%), which is a markedly better score than in the bacteria section. Still, while 82.8% of respondents reported brushing their own teeth regularly (vs. 76.5% of pregnant patients in London [55] and 68.9% of expectant mothers in Portugal [53]), only 62% knew that a baby’s gums should be cleaned even before any teeth erupt. This is consistent with earlier research reporting similar gaps in paediatric oral hygiene [4,35], although according to one study, four out of five participants knew of the need for gum cleaning [38].

Apart from identifying participants’ knowledge, another aim of the study was to find out if they knew when and how to obtain “information and services needed to make appropriate health decisions” [17]. Only one in five women knew when to take the child to their first dental check-up, which is consistent with some earlier findings [4,35] and, unfortunately, with reported practices [56]. It is a markedly lower proportion than that reported in a study from Croatia, in which 41% of women knew the right timing [57]. In addition, only 21% of our participants listed a dentist as a source from which they obtained paediatric OH information. For comparison, the main professional sources of information listed by Croatian women were a dentist (54%) or a gynaecologist (23%) [57]. Moreover, only 53.7% of our respondents obtained any paediatric OH information during or shortly before pregnancy, with only 5% mentioning antenatal classes, 11.5% obstetrician–gynaecologists, and 7.8% community midwives as their sources of knowledge. This is particularly notable given the fact that all the respondents were hospitalised. Hence, they probably had more interactions with health professionals than non-hospitalised women. The available literature suggests that the problem is not limited to inpatients: most pregnant women are not counselled on children’s OH and hygiene [4,58]. According to a recent study from the Małopolskie province, over 60% of expectant mothers had not received any professional oral hygiene instructions; two-thirds actually relied on internet sources of knowledge [36].

Insufficient availability of counselling was also reported (to a various extent) in other European countries. For example, according to the London study mentioned above, 65% of respondents received (any) oral health advice [55]. According to Bencze et al. (2021) [50], as of 2018, OH education for pregnant women was not available in 8 of the 27 European Union countries, 2 of which (Poland and Romania) had moderate to high DMFT indicators.

The fact that nearly all Polish women were estimated to be under gynaecological care during pregnancy [59,60] could be treated as an asset and an opportunity for gynaecologists to reach women with basic OH counselling or at least with a referral to a dentist before the woman chooses to visit a dentist (especially as the presented results and other research [34] suggest that only about half are likely to attend a dental office each half-year). Perhaps this unique position regarding gynaecologists is a reason why there are voices to increase the involvement of gynaecologists in the improvement of patients’ literacy, not only in the area of reproductive health [61,62] but also in OH [63,64,65]. According to the currently binding standard of perinatal care [66], a pregnant woman should be referred to a dentist during her first antenatal visit or up to the tenth week of pregnancy. Indeed, such referrals from gynaecologists were found to increase the chances of a woman attending a dental check-up during pregnancy by more than five times, and the chances were even higher when a gynaecologist requested dental consultation feedback about OH status [64]. This indicates that counselling provided by gynaecologists might serve as an impulse, guiding expectant mothers towards better OH literacy, and its use could be considered also in other countries with doctor-led maternity care models. To facilitate such a practice, it would be advisable to expand perinatal care standards so that they would include explicit guidelines as to when and how exactly such basic OH counselling should be performed.

At the same time, OH counselling needs to be available as part of easily accessible public health programmes and ought to be provided by persons working in various health professions [4,34,36,64], such as primary care midwives and general practitioners. Wider involvement of primary personnel could be a step towards building an integrated model of care, with OH care as its intrinsic part. Such tendencies can be seen: a new model of primary care with a bigger focus on disease prevention and coordinated care has just been piloted [67] and is expected to be launched [68] in Poland. In general, the need for integrating OH care with general healthcare has been noticed and investigated [69,70]. While it still requires more in-depth research, an important finding was that one of the most reported barriers to such integration was healthcare providers’ competencies [70]. Hence, it should be remembered that better OH counselling should only be provided by healthcare professionals who have received comprehensive training on how to deliver effective educational interventions [58,71].

### 4.2. Maternal Oral Health Behaviours and Outcomes

The present results suggest that despite the prevalent belief of being under dental care shared by 90.3% of respondents, only one-half of them (54.4%) have been to a dentist within the previous 6 months. It corroborates earlier research in which 53% of the surveyed women visited a dentist during pregnancy, and only 3% were referred to such a visit by a gynaecologist [47]. To compare, 26% of respondents from Croatia reported that they received such a referral [57]. Although one-third of our study group had at least once experienced a financial barrier to using dental care services, such perceptions did not turn out to be significant during pregnancy: the women who reported financial barriers did not differ from the other women in terms of the date of their last dental visit. It could then be hypothesised that pregnancy is the time when women try to stick to the schedule of recommended health appointments irrespective of economic obstacles. Pregnancy is, in fact, often referred to as a teachable moment in life [72] when “women are motivated to adopt healthy behaviour” [28]. Hence, if the participants sometimes failed to do that, it might be because of poor awareness of what it actually means to be under dental care and what kinds of services this care provides to expectant mothers. Another reason might be the fact that women who rely on the internet as their main source of OH information have certain misconceptions and fears of risks connected with using dental services during pregnancy [73,74,75], which is why they might be reluctant to use them often enough. Whichever of these reasons holds true, it points to the need for improving OH literacy among pregnant women.

In the study group, as many as 80.8% of women self-assessed their OH status as good or rather good. At the same time, 39.5% had experienced permanent tooth extractions due to caries from periodontal disease, and 32.5% mentioned pain as a reason for their last dental visit. Similarly, a national study on the OH of pregnant women found women’s self-assessments to be overly optimistic. While 14.7% of its participants perceived their OH negatively, dental check-ups revealed that only 15.2% did not require dental treatment, and around 70% with objectively poor OH were not aware of this fact [34]. Both the present and earlier findings seem to indicate that some pregnant women may erroneously assume themselves to have good OH and misunderstand what “good oral health” actually means, which is another argument for stressing the need to improve OHKL in this population.

### 4.3. Paediatric Oral Health Knowledge and Literacy vs. Demographics, Behaviours, and Outcomes

Similarly to the present findings, earlier research also indicated a link between knowledge and education levels [35,37], and between knowledge and having children [38,57]. Adults with poorer OHK and a lower capacity to understand health education may in fact have low OHL [8]. In light of the findings presented here, it seems that the subgroups of pregnant women with the highest risk of insufficient OHL include the youngest expectant mothers, those with the lowest levels of education, as well as those who report irregular toothbrushing, relatively worse OH in self-assessments, previous extractions, and pain as a reason for their last dental visits. However, considering the complexity of the presented relationships as well as the potential consequences of low parental OHL for the child [8], further research among pregnant women is recommended, at best preceded by developing a patient-friendly instrument for the measurement of OHKL that could be routinely used by health personnel. Such measurements would facilitate individualised health education and subsequent evaluation of its effectiveness, which expectant mothers urgently need irrespective of their place of residence, self-assessed economic status, or even frequency of dental visits.

### 4.4. Study Limitations

The study was based on self-reported data and the OH status of participants was not assessed during dental check-ups. Therefore, the results may be subject to recall bias or reflect participants’ reluctance to report behaviours or outcomes they considered inappropriate.

All the respondents were hospital inpatients, but their general health status was not analysed. In some cases, hospitalisation may have been due to imminent delivery. However, some respondents reported having pregnancy complications, which may have resulted from chronic health conditions. Health knowledge and literacy in certain populations of chronic patients may differ from healthy populations.

Study participants were recruited in only one province of Poland and the sample is not representative of the whole country.

The pregnancy trimester was not included in data analyses.

## 5. Conclusions

Given the fact that pregnancy, as a teachable moment, is likely to motivate expectant mothers to undertake beneficial behaviour changes, we propose an opportunistic approach to OH education and argue that counselling should be provided whenever and wherever an opportunity for education arises, by all healthcare professionals who come into contact with pregnant women. In all the countries where physician-led perinatal care coincides with scarce educational opportunities in dental offices, a special role could be played by gynaecologists. Primary care personnel are also in a position to provide basic OH counselling. In addition, hospitalised women should have a chance to obtain relevant information in hospitals.

To summarize the findings presented here, the efforts aimed at increasing oral health knowledge and literacy of expectant mothers should focus on:

1. The aetiology and infectious character of caries, together with methods of preventing infections;
2. The need for regular dental check-ups of expectant mothers and the appropriate timing of the first dental visit of the child;
3. The diagnosis of women’s OHKL by means of reliable tools, because their own beliefs regarding OH status and being under dental care may be overly optimistic and, as such, may not constitute the grounds for abandoning health education.

## Figures and Tables

**Table 1:** Characteristics of the study group.

Variable		*n*	%
Age (years)	<20	14	3.5
	20–29	189	47.3
	30–39	182	45.5
	≥40	15	3.8
Number of children	0 (This is the first pregnancy)	133	33.3
	1	147	36.8
	2	90	22.5
	3	21	5.3
	>3	9	2.3
Education	Primary	16	4.0
	Vocational	57	14.3
	Secondary	161	40.3
	Higher	166	41.5
Residence	Village	190	47.5
	Small town (<50 thousand inhabitants)	108	27.0
	Big city (≥50 thousand inhabitants)	102	25.5
Self-assessed economic status	Good	381	95.3
	Not good/bad	19	4.7
Economic status as a barrier to dental care	No	279	69.7
	Yes	121	30.3
Reported complications during present pregnancy	No	205	51.2
	Yes *	195	48.8
	Bleeding/spotting	56	14
	Hypertension	32	8
	Anaemia	31	7.8
	Diabetes	26	6.5
	Obstetric cholestasis	7	1.8
	Toxoplasmosis	6	1.5
	Other	52	13

* Note: A participant could report one or more complications.

**Table 2:** Reported maternal oral health behaviours and outcomes (*n* = 400).

Variable	*n*	%
**Behaviours**		
**Brushing teeth regularly** **(twice daily or after each meal)**		
Yes	327	81.8
No	73	18.2
**Using fluoride toothpaste**		
Yes	384	96
No	16	4
**Visiting a dentist at least once in the last 6 months**		
Yes	218	54.4
No	182	45.6
**Being under dental care**		
Yes	361	90.3
With one regular dentist	272	68
With casual dentists	89	22.3
No	39	9.2
**Outcomes**		
**Having healthy teeth (self-assessment)**		
Yes/rather yes	323	80.8
No/rather not/I don’t know	77	19.3
**Extractions of permanent teeth**		
No	242	60.5
Yes	158	39.5
**Pain as a reason for last dental appointment**		
No	270	67.5
Yes	130	32.5

**Table 3:** Paediatric oral health knowledge and literacy vs. demographics, behaviours, and reported outcomes.

Average Scores in the Respondent Subgroups (% of Correct Answers)						
Variable	OHK16 Total	OHK16 Bacteria Section (4 Questions)	OHK16 Substrate Section (5 Questions)	OHK16 Time Section (7 Questions)	OHL2 Understanding the Need for Services: Timing of First Dental Check-Up (Correct vs. Incorrect Responses)	OHL2 Seeking Information on Child’s Oral Hygiene (Yes vs. No)
**DEMOGRAPHICS**						
**Age**	*p* = 0.00404	*p* = 0.09300	*p* = 0.00251	*p* = 0.16750	*p* = 0.54026	*p* = 0.09656
<20	56.25%	39.29%	61.43%	62.24%	14.29%	42.86%
20–29	70.03%	58.68%	74.11%	73.61%	21.16%	48.15%
30–39	74.04%	60.99%	79.78%	77.39%	19.23%	60.44%
≥40	70.83%	65.00%	78.67%	68.57%	6.67%	53.33%
**Education**	*p* = 0.00001	*p* = 0.00001	*p* = 0.00018	*p* = 0.00000	*p* = 0.08932	*p* = 0.48466
higher	77.71%	65.66%	82.29%	81.33%	24.70%	56.63%
secondary	70.11%	57.92%	74.29%	74.09%	18.01%	53.42%
vocational	61.40%	49.12%	70.53%	61.90%	10.53%	50.88%
primary	54.69%	42.19%	58.75%	58.93%	12.50%	37.50%
**Self-assessed economic status**	*p* = 0.77348	*p* = 0.21079	*p* = 0.77738	*p* = 0.88216	*p* = 0.67574	*p* = 0.92020
good	71.75%	59.51%	76.69%	75.22%	19.69%	53.81%
not good/bad	64.47%	53.95%	71.58%	65.41%	15.79%	52.63%
**Economic status as a barrier to dental care**	*p* = 0.29939	*p* = 0.76044	*p* = 0.17043	*p* = 0.51258	*p* = 0.07000	*p* = 0.83357
no	71.21%	59.05%	76.20%	74.60%	21.86%	53.41%
yes	71.85%	59.71%	77.02%	75.09%	14.05%	54.55%
**Residence**	*p* = 0.27317	*p* = 0.22851	*p* = 0.41250	*p* = 0.66477	*p* = 0.06119	*p* = 0.19401
village	70.66%	57.11%	76.00%	74.59%	14.74%	50.53%
small town	72.28%	61.34%	78.15%	74.34%	22.22%	61.11%
big city	71.88%	61.03%	75.49%	75.49%	25.49%	51.96%
**Number of children**	*p* = 0.01956	*p* = 0.52223	*p* = 0.36139	*p* = 0.39580	*p* = 0.04825	*p* = 0.34290
0 (first pregnancy)	69.41%	56.02%	74.44%	73.47%	27.07%	48.12%
1	74.06%	63.44%	79.86%	76.00%	15.79%	54.14%
2	70.90%	58.06%	73.56%	76.35%	18.89%	57.78%
3	66.67%	52.38%	76.19%	68.03%	4.76%	57.14%
>3	73.61%	66.67%	80.00%	73.02%	11.11%	77.78%
**Having a preschool child**	*p* = 0.18759	*p* = 0.03190	*p* = 0.00561	*p* = 0.82402	*p* = 0.00410	*p* = 0.01058
yes	72.31%	61.35%	77.78%	74.67%	14.01%	59.90%
no	70.43%	56.99%	75.03%	74.83%	25.39%	47.15%
**BEHAVIOURS**						
**Brushing teeth regularly**	*p* = 0.00208	*p* = 0.00093	*p* = 0.00321	*p* = 0.01010	*p* = 0.08719	*p* = 0.01647
yes	73.11%	61.85%	77.86%	76.15%	21.10%	56.57%
no	63.78%	47.60%	70.14%	68.49%	12.33%	41.10%
**Using fluoride toothpaste**	*p* = 0.03886	*p* = 0.62043	*p* = 0.04344	*p* = 0.00025	*p* = 0.57090	*p* = 0.83781
yes	71.76%	59.24%	76.98%	75.19%	19.27%	53.65%
no	62.89%	59.38%	63.75%	64.29%	25.00%	56.25%
**Visiting a dentist at least once in the last 6 months**	*p* = 0.71358	*p* = 0.07016	*p* = 0.86458	*p* = 0.44575	*p* = 0.52802	*p* = 0.11507
yes	73.14%	62.39%	77.43%	76.21%	20.64%	57.34%
no	69.33%	55.49%	75.27%	73.00%	18.13%	49.45%
**Being under dental care**	*p* = 0.06813	*p* = 0.59236	*p* = 0.48986	*p* = 0.01624	*p* = 0.79688	*p* = 0.98989
yes	71.87%	59.70%	76.90%	75.23%	19.67%	53.74%
no	67.15%	55.13%	72.31%	70.33%	17.95%	53.85%
**OUTCOMES**						
**Having healthy teeth (self-assessment)**	*p* = 0.00137	*p* = 0.02219	*p* = 0.00035	*p* = 0.02780	*p* = 0.51894	*p* = 0.17058
yes/rather yes	72.95%	60.84%	77.96%	76.29%	20.12%	55.42%
no/rather not/I don’t know	64.94%	52.60%	70.13%	68.27%	16.88%	46.75%
**Extractions of permanent teeth**	*p* = 0.04613	*p* = 0.91771	*p* = 0.66485	*p* = 0.03136	*p* = 0.04378	*p* = 0.29784
no	72.08%	59.61%	76.36%	76.15%	22.73%	51.65%
yes	70.37%	58.70%	76.58%	72.60%	14.56%	56.96%
**Pain as a reason for the last dental appointment**	*p* = 0.01047	*p* = 0.02856	*p* = 0.49495	*p* = 0.16751	*p* = 0.79750	*p* = 0.18541
no	73.57%	62.55%	77.84%	76.82%	19.09%	56.43%
yes	68.12%	54.25%	74.34%	71.61%	20.13%	49.69%

**Table 4:** Participants’ characteristics vs. selected outcomes (multivariate logistic regression).

	Coefficient (β)	Standard Error for β	Wald χ^2^	*p*	Odds Ratio	95% CI for OR
**Having healthy teeth (self-assessment): YES**						
Intercept	−0.37	0.61				
Economic status as a barrier to dental care: YES ^1^	−0.91	0.28	10.6	<0.001	0.40	0.23 to 0.69
Being under dental care with one regular dentist ^2^	0.43	0.31	1.9	0.168	1.54	0.82 to 2.84
Being under dental care: NO ^2^	−0.96	0.43	5.0	0.025	0.38	0.16 to 0.89
OHK16 substrate section score	0.013	0.006	5.4	0.038	1.01	1.00 to 1.03
OHK16 time section score	0.014	0.007	4.0	0.038	1.01	1.00 to 1.03
**Extractions of permanent teeth: YES**						
Intercept	−2.47	0.69				
Residence: small town ^3^	−0.61	0.30	4.1	0.041	0.54	0.30 to 0.97
Residence: village ^3^	−0.49	0.27	3.3	0.065	0.61	0.36 to 1.03
Age	0.06	0.02	9.0	0.006	1.06	1.02 to 1.1
Education: primary ^4^	0.89	0.56	2.5	0.112	2.44	0.79 to 7.41
Education: secondary ^4^	0.57	0.25	5.2	0.021	1.77	1.09 to 2.88
Education: vocational ^4^	1.18	0.34	12.0	<0.001	3.27	1.7 to 6.39
Economic status as a barrier to dental care: YES ^1^	0.92	0.23	16.0	<0.001	2.51	1.60 to 3.96
**Pain as a reason for last dental appointment: YES**						
Intercept	0.36	0.49				
Education: primary ^4^	1.69	0.60	8.0	0.004	5.43	1.75 to 18.94
Education: secondary ^4^	0.24	0.26	0.7	0.35	1.27	0.77 to 2.12
Education: vocational ^4^	0.95	0.34	7.7	0.006	2.59	1.32 to 5.10
Economic status as a barrier to dental care: YES ^1^	0.35	0.24	2.1	0.147	1.42	0.88 to 2.30
Being under dental care with one regular dentist ^2^	−0.94	0.27	12.4	<0.001	0.39	0.23 to 0.66
Being under dental care: NO ^2^	−0.12	0.40	0.09	0.767	0.89	0.40 to 1.95
OHK16 bacteria section score	−0.01	0.004	4.6	0.031	0.99	0.98 to 0.99

Reference categories for categorical variables: ^1^ economic status as a barrier to dental care: NO; ^2^ being under dental care with casual dentists; ^3^ residence: big city; ^4^ education: higher.

## Data Availability

The datasets generated and analysed during the current study are available from the corresponding author.

## Abbreviations

OH: oral health, OHL: oral health literacy, OHK: oral health knowledge, OHKL: oral health literacy and knowledge.
